# Body measurements correlation and x-ray imaging of three *Hystrix javanica* fetuses in different fetal development stages

**DOI:** 10.1590/1984-3143-AR2021-0005

**Published:** 2021-08-25

**Authors:** Yosua Kristian Adi, Surya Agus Prihatno, Irma Padeta, Teguh Budipitojo

**Affiliations:** 1 Department of Reproduction and Obstetric, Faculty of Veterinary Medicine, Universitas Gadjah Mada, Yogyakarta, Indonesia; 2 Department of Anatomy, Faculty of Veterinary Medicine, Universitas Gadjah Mada, Yogyakarta, Indonesia

**Keywords:** linear regression, measurements, morphology, radiography, sunda porcupine fetus

## Abstract

*Hystrix javanica* is endemic species in Indonesia. Study about fetal development of *Hystrix javanica* are very rare because of sample limitation. This study was carried out to describe the morphometrics and x-ray analysis of three fetuses in different stage to give basic information about fetal development of *Hystrix javanica*. Three fetus samples fixed in Bouin’s solution was used in this study. Observation was carried out to identify the characteristic of three fetus samples. This included the pattern of hair, body measurements, body volume, and body weight. X-ray analysis was carried out to know the ossification process in the fetal development. Statistical analysis was carried out using Microsoft 365® Excel program software. Three fetus samples had different specific hair pattern, that was hairless, smooth hairs, and smooth hairs with dense-non dense pattern. Body volume of 1^st^, 2^nd^, and 3^rd^ fetus were 23mL, 90mL, and 170mL, respectively. Body weight of 1^st^, 2^nd^, and 3^rd^ fetus were 19.5g, 79.22g, and 153.18g, respectively. Pearson’s correlation analysis shown strong relationship between total body length, front body length, back body length, horizontal body diameter, vertical body diameter, head length, and head diameter against body volume and body weight of three fetuses. Significant positive correlation was shown between horizontal body diameter, vertical body diameter, and head diameter against body volume and body length with P value < 0.05. Faint radiopaque images showed in the 2^nd^ fetus sample and strong radiopaque images showed in the 3^rd^ fetus sample. Radiopaque images were identified in the teeth, cranium, vertebrae, and extremities bones. In this study we concluded that there was a specific hair pattern in different fetal stage. All body measurements have positive correlation with body volume and body weight and x-ray analysis shown that the ossification of the bone was started to happen while the smooth hair was growth.

## Introduction 

Animal that typically only found in defined geographic area, or called endemic species, are characteristic elements of local biodiversity (Behroozian et al., 2020). So that, endemic species become interesting object material for research because of its unique characteristic. Sunda porcupine, mammals belong to Hystricidae, is big rodent that can be found only in Indonesia (Aplin, 2016). This species has Latin name *Hystrix javanica*. In 2016, the International Union for Conservation of Natural Resources (IUCN) still classified this species into Least Concern which means the population in nature is still abundant. However, Indonesian Government has classified Sunda porcupine into a protected animal since 2018. Mustikasari et al. (2019) reported that this species still could be observed in the designated area of Cisokan Hydropower, West Java, Indonesia. This is good news because the other species belongs to Hystricidae such as Malayan porcupine (*Hystrix brachyura*) is known to be lack of sightings from 1970‒2000 in their natural habitats, suggested that the species was either locally rare or extinct (Chung et al., 2016).

Embryonic and fetal development of Sunda porcupine are very rarely studied because of sample limitation. This species is not yet mass bred, so the study about reproductive aspect will encountered difficulty in obtaining the samples. Collaboration with local hunting communities that hunted this species for subsistence purposes is one option. This can be the alternate to collect in situ data and biological samples with a higher level of reliability (Mayor et al., 2017). Since wildlife still become a significant source of food and financial resource for rural and urban populations in many Indonesian regions, obtaining the porcupine samples are still possible. Most people hunt the porcupine for meat consumption. However, the high rate of exploitation for some species will reduce their population in nature, which in turn increases its value and leads to its extinction in the wild (Hall et al., 2008).

In a tropical country, such as Indonesia, the reproductive activities of porcupine, especially in captive breeding usually occur in the late summer and the early rainy season. The gestation period of most members of the genus Hystrix is 100 to 110 days. The litter comprises one or two precocial (Nowak, 1964) and most rodent species produce altricial neonates (Derrickson, 1992). However, the knowledge about reproduction of Sunda porcupine, especially embryonic and fetal development still not available. The information or data about morphological change of Sunda porcupine fetuses during gestation is very important to monitor the pregnancy.

## Methods

### Ethical approval

We have arranged this study according to the laboratory protocol for research in Universitas Gadjah Mada. All the materials and methods that have been used in this study to collect the data has been approved by the Ethics Committee of Universitas Gadjah Mada, Indonesia with No.326/KEC-PPT/IX/2015.

### Sample collection

Three fetus samples were obtained from slaughterhouse in Tawangmangu, Central of Java, Indonesia in 2017. Soon, after the fetus was remove from body cavity, it was fixed with Bouin’s solution for 24 hours. After that, the solution was changed with 70% alcohol solution for preservation.

### Body parameter

Observation was carried out to identify the characteristic of three fetus samples. This included the pattern of hair, body measurements, body volume, and body weight. Parameter for body measurements were total body length, front body length, back body length, horizontal body diameter, vertical body diameter, head length, and head diameter. Total body length, front body length, and back body length were measured with thin rope that laid along the dorsal surface from nose to the end of tail. The mid body landmark was determined by the change of specific skin pattern. Horizontal body diameter, vertical body diameter, head length, and head diameter were measured with vernier caliper (Tricle Brand^®^). Body volume was calculated using the Archimedes’ principle and body weight was measured using weigher (model: EHA401, CAMRY^®^).

### X-ray analysis

Radiography test was done using x-ray camera (XM-F50-100 ii 50mA). Three fetus samples were placed laterally lay on the radiographic film and took the photograph. Radiographic film was digitally processed then the picture was saved as PDF file. Image processing was carried out using hardware and software from FCR PRIMA V, FUJIFILM^®^. The result was analyzed descriptively to know the ossification process of the fetal bone by identified the radiopaque and radiolucent images.

### Statistical analysis

The data of body measurements was statistically analyzed for correlation with body volume and body weight. Correlation between body measurements against body volume and body weight of three fetus samples were measured with Pearson’s correlation analysis. Analysis was carried out with Microsoft 365® Excel program software. Linear regression analysis was performed in this study to show the regression coefficient and regression equation.

## Results

**Figure 1:**
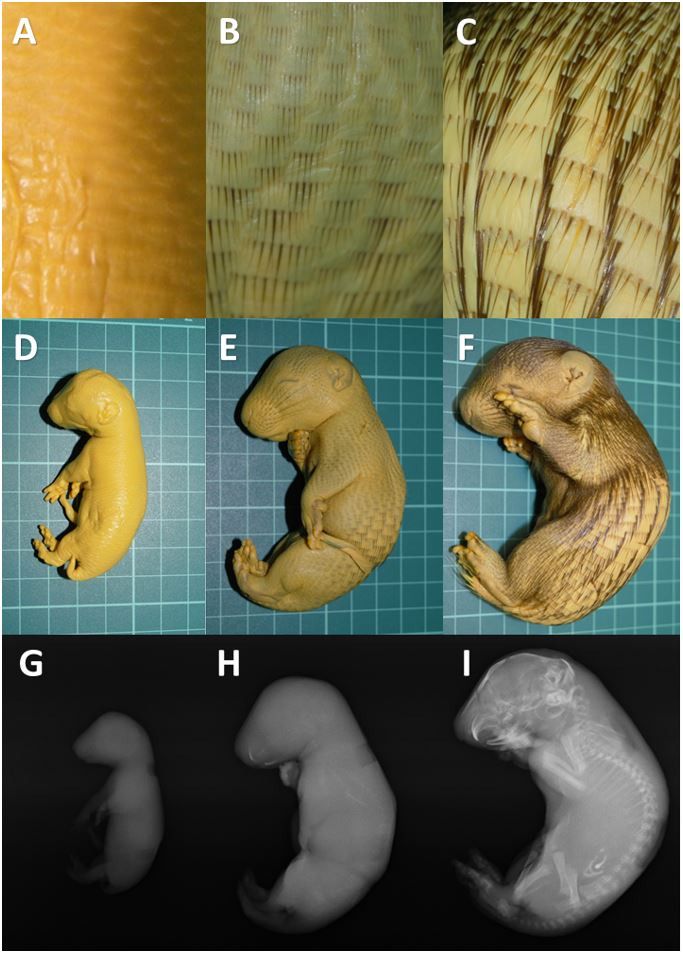
Hair pattern, fetus morphology, and x-ray imaging of three *Hystrix javanica* fetuses.

Three fetus samples had specific hair pattern, respectively. The smallest one, the 1^st^ fetus, was hairless (Figure 1 A, D). From our observation and analysis, 1^st^ fetus sample has total body length 12.13 cm, front body length 6.93 cm, back body length 5.35 cm, horizontal body diameter 1.99 cm, vertical body diameter 2.07 cm, head length 2.85 cm, head diameter 1.83 cm, body volume 23 mL, and body weight 19.5 g (Table 1). The external organogenesis almost completed. The four limbs have perfectly formed. However, ears and eyes canals were not clearly observed. In the medium one, the 2^nd^ fetus, smooth hairs were observed in all the body surface (Figure 1B, E). From our observation and analysis, 2^nd^ fetus sample has total body length 19.83 cm, front body length 10.87 cm, back body length 9.13 cm, horizontal body diameter 3.59 cm, vertical body diameter 3.5 cm, head length 4.53 cm, head diameter 2.82 cm, body volume 90 mL, and body weight 79.22 g (Table1). Further development of external organs could be observed clearly. In the biggest one, the 3^rd^ fetus, we could clearly differentiate two pattern of body hair (Figure 1C, F). In the front half of the body, the hair densely arranged, while in the back half of the body, the hair was arranged in a dense-non dense pattern that extends towards the back. From our observation and analysis, 3^rd^fetus sample has total body length 23.47 cm, front body length 12.2 cm, back body length 11.22 cm, horizontal body diameter 5.06 cm, vertical body diameter 4.65 cm, head length 5.26 cm, head diameter 3.54 cm, body volume 170 mL, and body weight 153.18 g (Table 1).

**Tables 1:** Body measurements, body volume, and body weight data of three fetus samples.

**Parameter**		**1^st^ Fetus**	**2^nd^ Fetus**	**3^rd^ Fetus**
Body Measurements (cm)	Total Body Length	12.13	19.83	23.47
	Front body length	6.93	10.87	12.2
	Back body length	5.35	9.13	11.22
	Horizontal body diameter	1.99	3.59	5.06
	Vertical body diameter	2.07	3.5	4.65
	Head length	2.85	4.53	5.26
	Head diameter	1.83	2.82	3.54
Body Volume (mL)		23	90	170
Body Weight (g)		19.5	79.22	153.18

**Figure 2:**
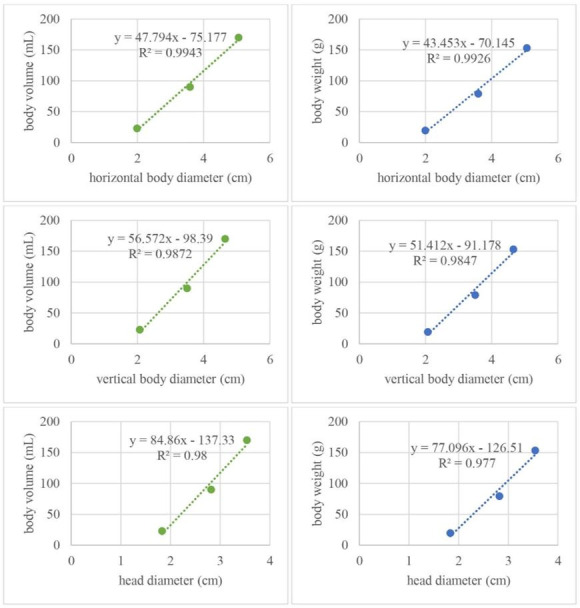
Linear regression analysis of body parameters against body volume and body weight.

Pearson’s correlation analysis of body measurements against body volume and body weight shown strong relationship with correlation coefficient (r) > 0.9. Based on our statistical analysis, the value of correlation coefficient (r) of total body length, front body length, back body length, horizontal body diameter, vertical body diameter, head length, and head diameter against body volume were 0.968; 0.946; 0.977; 0.997 (P < 0.05); 0.994 (P < 0.05); 0.962; and 0.99 (P < 0.05), respectively (Table 2). Significant positive correlation was shown between body volume against horizontal body diameter, vertical body diameter, and head diameter with P value < 0.05. Therefore, the strongest correlation with body volume was horizontal body diameter with (r) value 0.997. The value of correlation coefficient (r) of total body length, front body length, back body length, horizontal body diameter, vertical body diameter, head length, and head diameter against body weight were 0.965; 0.943; 0.975; 0.996 (P < 0.05); 0.992 (P < 0.05); 0.96; and 0.988 (P < 0.05), respectively (Table 2). Significant positive correlation was shown between body length against horizontal body diameter, vertical body diameter, and head diameter with P value < 0.05. The strongest correlation with body weight was same with the strongest correlation with body volume that its horizontal body diameter with r value 0.996. Linear regression analysis that performed for significant positive correlation parameter against body volume and body weight was shown in Figure 2.

**Tables 2:** Correlation coefficient (r) of body measurements against body volume and body weight.

**Correlation coefficient (r)**		**Body volume**	**Body weight**
Body measurements (cm)	Total body length	0.968	0.965
	Front body length	0.946	0.943
	Back body length	0.977	0.975
	Horizontal body diameter	0.997*	0.996*
	Vertical body diameter	0.994*	0.992*
	Head length	0.962	0.960
	Head diameter	0.990*	0.988*

* P < 0.05

Radiography test using x-ray analysis shown different radiopaque and radiolucent images in three fetus samples. In the 1^st^ fetus, there was no radiopaque images that could be identified (Figure 1G). Only radiolucent interpretation was observed. In the 2^nd^ fetus, radiopaque images could be identified clearly only in the teeth (Figure 1H). Radiopaque images in the ossification of vertebrae and other bone were faint. In the 3^rd^ fetus, radiopaque images were strongly observed in the teeth, cranium, vertebrae, and extremities bones (Figure 1I).

## Discussion

In captivity, male Sunda porcupine reaches sexual maturity at age 9-12 month, while female Sunda porcupine at age 12 month. Gestation period of Sunda porcupine is about 100-112 days. Neonates of Sunda porcupine has short and soft hair or quills (Inayah et al., 2020). Hair covering the body at birth also found in other rodent species such as the red-rumped agouti (*Dasyprocta leporina Linnaeus*, 1758) (Oliveira et al., 2017), the lowland paca (Cuniculus paca) (El Bizri et al., 2017), the agouti (*Dasyprocta prymnolopha*) (Fortes et al., 2013), the coypu (*Myocastor coypus*) (Felipe and Masson, 2008), and the Guinea pig (*Cavia porcellus*) (Evans and Sack, 1973). However, small-sized rodent produces less-developed neonates such as the rat, the mice, and the coruro that were born hairless (Kaufman, 1992; Bisaz and Sullivan, 2012; Theiler, 1989; Burda 1989; Begall et al., 1999). In this study, hairless fetus was identified only in the 1^st^ fetus sample, while 2^nd^ and 3^rd^ fetus samples were haired.

Inayah et al. (2020) reported that the weight at birth of Sunda porcupine neonates was approximately 232.9 g with the length of head, body, and tail were 6 cm, 15 cm, and 5 cm, respectively or 26 cm in total. Our biggest fetus sample (3^rd^ fetus sample) was 153.18 g in weight and 23.47 cm in total length. In domestic animal, such as cattle, known that the highest growth rate occurs in third trimester period of gestation with the increase of body weight in third trimester is about 3-fold greater than the increase in second trimester (Mao et al., 2008). We guessed that our three fetus samples were in second or third trimester of gestation. In mouse and rat, 1^st^ fetal stage, characterized by rapid growth of eyelids (eyes entirely covered at end of 18th day); palate complete; pinna covers ear duct; umbilical hernia withdraws is occurred at 17-18 day gestation from 19 and 22 day of gestation period. The length of mouse and rat fetuses in the 1^st^ fetal stage is 16-20 mm, while the length at birth is 20-40 mm (Witschi, 1962). Our smallest sample, 1^st^ fetus sample, showed eyes that entirely covered by eyelids and pinna has been formed. Total body length was 12.13 cm or approximately a half of body length at birth. We predicted that the 1^st^ fetus sample was at early 1^st^ fetus stage. Unfortunately, we could not get data of the gestational age from the three fetus samples clearly.

Very strong relationship between body measurements against body volume and body weight was identified using Pearson’s correlation analysis. Body diameter and head diameter have higher correlation against body volume and body weight than the other parameters. These parameters are commonly used to predict gestation age and delivery date during pregnancy in dogs and cats (Luvoni and Grioni, 2000; Beccaglia et al., 2016). In addition, head diameter with maternal pelvic diameter also commonly used to predict risk of dystocia, due to oversized fetus or relatively small pelvic canal (Limmanont et al., 2019).

The organogenesis and differentiation of the skeleton take place during both embryonic and fetal stages, while maturation continues during postnatal life (Garbis-Berkvens and Peters, 1991). Radiologic visualization, x-ray analysis, can be used to identify the earlier developing embryonic skeleton (van Zalen-Sprock et al., 1997). In this study, we found that in the 1^st^ fetus sample, the ossification was not developed yet. This proved with no-radiopaque imaging. In the 2^nd^ fetus sample, radiopaque images were identified in the teeth, cranium, and vertebrae. Although the radiopaque images on the cranium and vertebrae were still faint and not as clear as on the teeth. In rat, the first ossification centers are seen in the clavicula, ribs and some bones of the skull on the 16^th^ day of gestation. Head grows rapidly and the palate drafts fuse nn the 18th day, while the spaces of the finger joints of the anterior and posterior extremities are still seen on the 19th and 20th days (Yilmaz and Tokpınar, 2019). In the 3^rd^ fetus sample, ossification was seen clearly in the skull, vertebrae, ribs, long bone of extremities, and coxae. This indicated that the fetus was in the late stage of ossification and almost ready to birth.

## Conclusion

Three skin-hair patterns of Sunda porcupine fetus have been identified in this study. All body measurements have correlation with body volume and body weight of Sunda porcupine fetus, with significant positive correlation was found in horizontal body diameter, vertical body diameter, and head diameter. X-ray analysis shown the ossification started to happen in 2^nd^fetus sample and almost completed in 3^rd^ fetus sample. Further research on the estimated day of pregnancy in Sunda porcupine in captivity needs to be done using ultrasonography, since data collection of fetuses from nature is very difficult and almost impossible at this time. This is important for the conservation of this species in the future.
